# Effect of Copper Acyclovir Complexes on Herpes Simplex Virus Type 1 and Type 2 (HSV-1, HSV-2) Infection in Cultured Cells

**DOI:** 10.1155/MBD.1998.19

**Published:** 1998

**Authors:** M. Panteva, T. Varadinova, I. Turel

**Affiliations:** 1 Laboratory of Virology Faculty of Biology Sofia University 8 Dragan Tzankov Blvd. Sofia 1421 Bulgaria; 2 University of Ljubljana Faculty of Chemistry and Chemical Technology Askerceva 5 Ljubljana 1000 Slovenia

## Abstract

We have found that when copper, zinc or cobalt is bound to a suitable ligand, the appropriate complex exhibited a significant anti-HSV effect (Varadinova et al., 1993; 1996). Recently published data by Sagripanti et al. (1997) also show that the inhibition of HSV by copper was enhanced by reducing agents and that mechanism of the inactivation is similar as for copper-mediated DNA damage (Aruoma, et al. 1991; Dizdaroglu, et al., 1991; Toyokuni and Sagripanti, 1994). Therefore it was interesting to study the efect of Cu(ll) coordination compounds with acyclovir (ACV) on the replication of HSV in cultured cells. The experiments on cytotoxicity as well as on the activity of three different Cu-ACV complexes [Cu(ACV)_2_Cl_2_(H_2_O)_2_] = (A); [Cu(ACV)_2_(H_2_O)_3_](NO_3_)_2_.H_2_O = (B) and [Cu(ACV)_2_(H_2_O)_2_](NO_3_)_2_] = (C) towards virus replication, with special attention on the growth of ACV-resistant strain R-100 were performed on MDBK cells. ACV was used as a reference compound. The following results were obtained: 1) Increased cell’s viability in the presence of 20-40(g/ml ACV and decreased one in the presence of Cu-ACV complexes with relative level (A) >> (B) > (C); 2) Cu-ACV complexes are more cytotoxic than the ligand - ACV and the relative level is (C)>(B)>(A); 3) The anti-HSV effect of ACV can be modulated by copper at levels depending on the specificity of the particular virus strain: (i) for the ACV sensitive strain DA (HSV-1) - ACV ((A) > (C) > (B); (ii) for the ACV sensitive strain Bja (HSV-2) (A) > ACV > (C) > (B); (iii) for strain R-100 (ACV^R^, TK^a^) - (A) > ACV > (C) > (B). This findings are consistent with previously published data and undoubtedly show that Cu-ACV complexes could be useful in the treatment of HSV infections, especially when the causative agent is a resistant to ACV mutant.


### EFFECT OF COPPER ACYCLOVIR COMPLEXES ON HERPES SIMPLEX VIRUS TYPE 1 AND TYPE 2 (HSV-1, HSV-2) INFECTION IN CULTURED CELLS

M. Panteva[1], T. Varadinova[1], and I. Turel[2]

[1] Laboratory of Virology, Faculty of Biology, Sofia University, 8 Dragan Tzankov Blvd., 1421 Sofia, Bulgaria
[2] University of Ljubljana, Faculty of Chemistry and Chemical Technology, Askerceva 5, 1000 Ljubljana, Slovenia

**Abstract**
We have found that when copper, zinc or cobalt is bound to a suitable ligand, the appropriate complex exhibited a significant anti-HSV effect (Varadinova et al., 1993; 1996). Recently published data by Sagripanti et al. (1997) also show that the inhibition of HSV by copper was enhanced by reducing agents and that mechanism of the inactivation is similar as for copper-mediated DNA damage (Aruoma, et al. 1991; Dizdaroglu, et al., 1991; Toyokuni and Sagripanti, 1994). Therefore it was interesting to study the efect of Cu(II) coordination compounds with acyclovir (ACV) on the replication of HSV in cultured cells. The experiments on cytotoxicity as well as on the activity of three different Cu-ACV complexes $[Cu(ACV)_2Cl_2(H_2O)_2]$ = (A); $[Cu(ACV)_2(H_2O)_3](NO_3)_2.H_2O$ = (B) and $[Cu(ACV)_2(H_2O)_2](NO_3)_2]$ = (C) towards virus replication, with special attention on the growth of ACV-resistant strain R-100 were performed on MDBK cells. ACV was used as a reference compound. The following results were obtained: 1) Increased cell's viability in the presence of 20-40(g/ml ACV and decreased one in the presence of Cu-ACV complexes with relative level (A) >> (B) > (C); 2) Cu-ACV complexes are more cytotoxic than the ligand - ACV and the relative level is (C)>(B)>(A); 3) The anti-HSV effect of ACV can be modulated by copper at levels depending on the specificity of the particular virus strain: (i) for the ACV sensitive strain DA (HSV-1) - ACV ( (A) > (C) > (B); (ii) for the ACV sensitive strain Bja (HSV-2) - (A) > ACV > (C) > (B); (iii) for strain R-100 ($ACV^R$, $TK^a$) - (A) > ACV > (C) > (B). This findings are consistent with previously published data and undoubtedly show that Cu-ACV complexes could be useful in the treatment of HSV infections, especially when the causative agent is a resistant to ACV mutant.

**Key words**: Herpes simplex virus, acyclovir-ACV, ACV-resistant mutant, copper, inhibition.

**Introduction**
Acyclovir (ACV), the acyclic analogue of deoxyguanosine (9-(2-hydroxyethoxymethyl)guanine), is known as a specific and selective inhibitor of the replication of herpes viruses (Elion et al., 1977). ACV has been effectively used over a decade in the treatment of patients with herpes simplex and herpes zoster diseases (Tilson et al., 1993). Entering the infected cells, ACV is rapidly phosphorylated to ACV-monophosphate (ACV-MP) by the herpes virus-induced thymidine kinase (TK) (Elion et al., 1977) and subsequently to ACV-diphosphate (ACV-DP) and ACV-triphosphate (ACV-TP) by host-cell kinases (Elion et al., 1977; Fyfe et al., 1978). The ACV-TP functions as a specific substrate for viral DNA polymerase (Elion et al., 1977; Schaeffer et al., 1978) thus preventing both the formation of the DNA replication complex and the elongation of the DNA chain (Furman et al., 1984, Reardon and Spector, 1989; Elion, 1993).
However, the multiple use of ACV, especially in suboptimal doses, leads to formation or selection of ACV-resistant viral mutants, a problem that is increasingly common (Chatis and Grumpacker, 1992). This fact has encouraged the efforts to develop new drugs using, in particular, complexes of essential metals like copper, zinc and cobalt with different ligands. As some of the complexes tested significantly inhibited HSV growth (Varadinova et al., 1996; Sagripanti et al., 1997), we venture to propose that it is possible to increase the anti-HSV activity of ACV by complexing Cu(II), with special attention on the action towards replication of ACV-resistant mutant. For this purpose we used three coordination compounds in which the roles of Cu(II) and ACV are distinctively different - $[Cu(ACV)_2Cl_2(H_2O)_2]$; $Cu(ACV)_2(H_2O)_3](NO_3)_2.H_2O$ and $[Cu(ACV)_2(H_2O)_2](NO_3)_2]$.

### Materials and Methods

***Viruses and cells***. The two laboratory strains, DA (HSV-1) and Bja (HSV-2), were kindly provided by Prof. S. Dundarov (NCIPD, Bulgaria). The strain R-100, which is resistant to ACV and has an altered TK substrate specificity ($ACV^R$, $TK^a$), was kindly supplied by Prof. G. Palu (Institute of Microbiology, University of Padova, Italy). Madin-Darby bovine kidney (MDBK) cells were cultured at 37(C as monolayers in RPMI-1640 medium (Flow Laboratories, USA) supplemented with antibiotics (penicilin and streptomycin) and 10% bovine serum (NCIPD, Bulgaria). Serum concentration was reduced to 5% for growth of viruses and for testing the complexes.

***Complexes***. The following three complexes of Cu(II) with ACV were tested: $[Cu(ACV)_2Cl_2(H_2O)_2]$ = (A); $[Cu(ACV)_2(H_2O)_3](NO_3)_2.H_2O$ = (B) and $[Cu(ACV)_2(H_2O)_2](NO_3)_2]$ = (C). The complexes were synthesized as is reported elsewhere (Blazic et al., 1993; Turel et al., 1997; Turel et al., in preparation). The complexes were dissolved in DMSO and the stock solutions in distilled water were stored at +4(C. The solutions in growth medium used in the experiments were prepared *ex tempore*. ACV was used as a reference compound and its solutions were prepared as described above.

***Cytotoxicity assay – determination of the maximal nontoxic concentration (MNC) and the effect on growth kinetics and cell viability.*** To compare the MNC values of Cu-ACV complexes to that of ACV confluent monolayers were covered with media modified with 40, 30 or 20(g/ml from the appropriate complex and cultured at 37(C for 96h. Samples of cells grown in test complex-free medium served as a control. The maximal concentration, which did not alter neither the morphology nor the viability of the cells, was recognised as MNC. The effect of Cu-ACV complexes on growth kinetics and cell viability was evaluated on confluent monolayers, cultured in media modified with the appropriate complex in the same concentration range as for MNC test. Cells grown in ACV modified medium, as well as in umnodified one served as controls. On the 24, 48, 72 and 96h cells from monolayers were suspended using trypsin-EDTA and stained with 0.4% trypan blue in water for 15min. The total number of cells per sample, as well as the amount of viable cells, was counted using Burker's camera. The viability was calculated as a per cent from the total number of cells per sample. Each experiment was done in duplicate.

***Effect of complexes on the replication of HSV.*** Experiments were done in multicycle growth conditions. Confluent cell monolayers were washed and infected with 100 cell culture infectious doses (CCID) of the appropriate virus strain. After 1h for adsorption, cells were covered with maintenance media modified with test complexes in concentrations as above. One set of infected cells served as untreated control. The effect on viral replication was determined after 48h (for strains DA and Bja) and 72h (for R-100 strain) culturing at 37(C by reduction of infectious virus titres as compared to that of untreated viral control. The 50% inhibitory concentration ($IC_{50}$) for virus-induced cytopathic effect (CPE) was determined by a dose-response curve. To calculate the standard deviation of $IC_{50}$, each experiment was done in triplicate (for HSV-1 strain DA) or duplicate (for HSV-1 strain R-100 and HSV-2 strain Bja) and the Student's *t*-test was used.

Table 1. MNC values of Cu - ACV complexes

| Complex[a] | Toxicity | MNC[a] |
|---|---|---|
| A | | |
| 40 | +/- | |
| 30 | - | 30 |
| 20 | - | |
| B | | |
| 40 | + | |
| 30 | +/- | 20 |
| 20 | - | |
| C | | |
| 40 | + | |
| 30 | +/- | 20 |
| 20 | - | |

[a] in (g/ml; (+) cytotoxic effect; (-) lack of toxicity

### Results

***Cytotoxicity assay.*** Microscopically the MNC for Cu-ACV complexes was determined as 30(g/ml for (A) and 20(g/ml for (B) and (C) (Table 1). Additional data were found when the viability of cells was studied. As shown in fig. 1D, the number of viable MDBK cells cultured in ACV-modified medium was increased during the whole period of investigation as compared to that of untreated control. Moreover, the viability was significantly increased when the concentration of ACV was decreased. This is well manifested after 48 and 72h. In contrast, all three Cu-ACV complexes tested were more toxic for MDBK cells than ACV itself (fig. 1A-C). The concentrations that did not affect the cell viability were determined as 20(g/ml for (B) and (C) and 30(g/ml for (A) (this confirm once again the results from microscopical observation). No stimulation of the viability was detected.

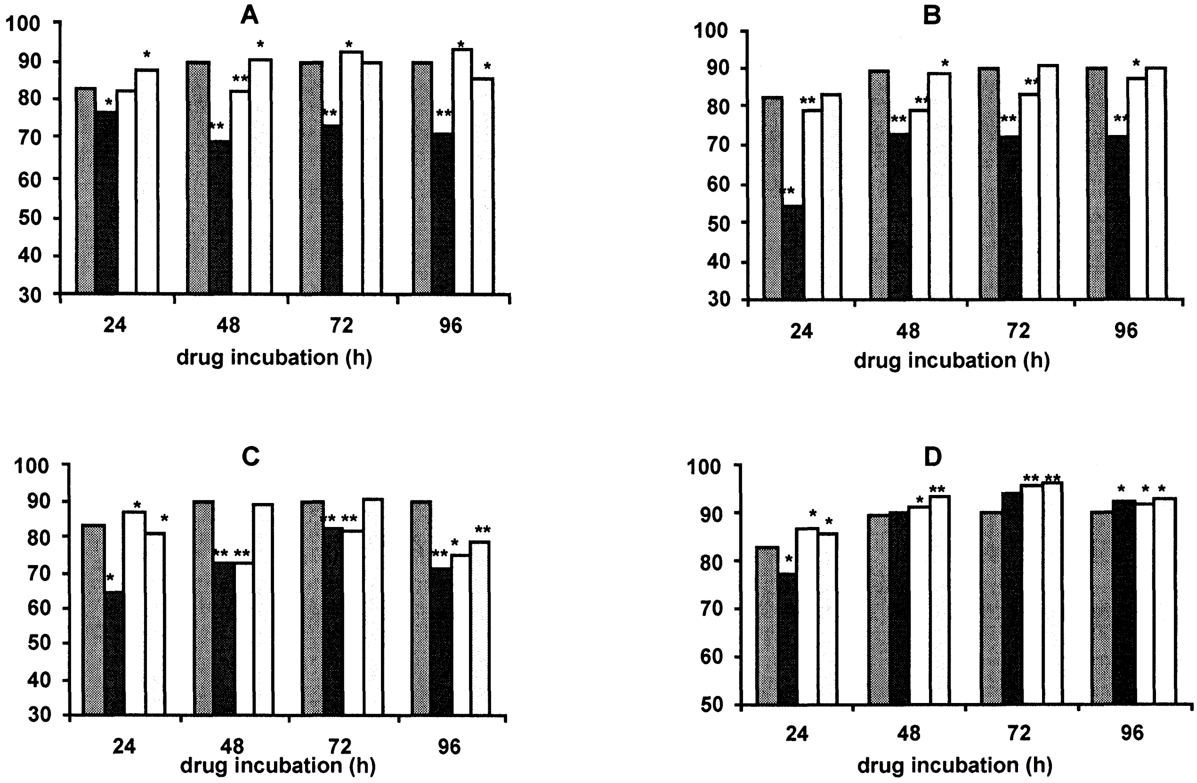

Fig. 1. Effect of Cu - ACV complexes (A, B and C) and ACV (D) on cell viability (*p<0.5; **p<0.05).

***Antiviral activities of Cu-ACV complexes.*** As shown in Table 2, ACV had substantial antiviral activity against the laboratory viral strains used. The effect of ACV on HSV growth was dependent on the viral type. Thus, HSV-1strain DA was up to 3.5 times more sensitive to ACV than HSV-2 strain Bja. As the strain R-100 is resistant to ACV, the $IC_{50}$ value was 40 times higher than that of strain DA.

Table 2. Antiviral efficacy of Cu-ACV complexes and ACV against HSV

| Virus (strain) | $IC_{50}$ ((g/ml)[a] | | | |
|---|---|---|---|---|
| | A | B | C | ACV |
| HSV-1 (DA)[b] | 0.79 ( 0.09* | 2.1 ( 0.47** | 1.42 ( 0.78* | 0.68 ( 0.2 |
| HSV-1 (R-100)[c] | 23.04 ( 0.89** | 26.92 ( 0.36* | 26.7 ( 2.15 | 26.19 ( 0.12 |
| HSV-2 (Bja)[c] | 1.44 ( 0.41* | 3.33 ( 0.34* | 2.97 ( 0.06* | 2.49 ( 0.17 |

[a] concentration of complex at which virus-induced cytopathic effect was reduced to a half of that in the viral control
[b] mean ( s.d. (three experiments); [c] mean ( s.d. (two experiments)
* p< 0.5; **p< 0.05

Among all three Cu-ACV complexes, only the one containing chloride (A) was more active to HSV-2 and the $IC_{50}$ value (1.44(g/ml) was significantly lower than that of ACV itself (p<0.5). Moreover, the complex (A) was also significantly effective (p<0.05) against the ACV - resistant virus R-100 when $IC_{50}$ was 23.04(g/ml.
The other two Cu-ACV complexes, (B) and (C), in which chloride is replaced by nitrate(V) ions, were less effective against the replication of the all three viral strains tested. However, the complex (C), in which a pseudo-chelate N-7/O-6 bonding of ACV to Cu(II) was observed, was more active against HSV replication than complex (B) in which a pseudo-chelate was not found.

The summarized data show that the anti-HSV effect of ACV can be modulated by copper at relative levels depending on the specificity of the particular virus strain: (i) for the ACV sensitive strain DA (HSV-1) - ACV ( (A) > (C) > (B); (ii) for the the ACV sensitive strain Bja (HSV-2) - (A) > ACV > (C) > (B); (iii) for strain R-100 ($ACV^R$, $Tk^a$) - (A) > ACV > (C) > (B). Concerning the cell viability the effect of Cu-ACV complexes followed the relative level: (A) >> (B) > (C).

**Discussion**

We have found that when copper, zinc or cobalt is bound to a suitable ligand, the appropriate complex exibited a significant anti-HSV effect (Varadinova et al., 1993; 1996). Recently published data by Sagripanti et al. (1997) also show that the inhibition of HSV by copper was enhanced by reducing agents and that mechanism of the inactivation is similar as for copper-mediated DNA damage (Aruoma, et al. 1991; Dizdaroglu, et al., 1991; Toyokuni and Sagripanti, 1994). These findings reveal that ACV-coordination compounds could also be potent antiviral drugs. In order to check this we used three types of Cu-ACV complexes, (A), (B) and (C). Their crystal structures prove that the roles of metal and ACV are different (Blazic et al., 1993; Turel et al., 1997; Turel et al., in preparation). The only similarity among the structures of all three complexes is that two ACV molecules are bound with Cu(II) through N-7.

Firstly, to elucidate the specific anti-viral effect we had to determine the cytotoxic range of Cu-ACV complexes and to compare it with that of the ligand, ACV. All three Cu-ACV complexes were more cytotoxic than the ligand - ACV, for which the $CC_{50}$ values vary considerably among different cell lines (up to 260(g/ml) (De Clerq, 1982; Collins, 1983). This is probably because each complex contains two molecules of ACV. It is also possible that complexes are able to dissociate and free ions like Cu(II), $Cl^-$ or $NO_3^-$ could express additional cytotoxic effect. This was concluded when the precise crystal structures were analysed. Thus, in the complex (A) Cu(II) atom is bound to two *trans*-positioned ACV ligands monodentately through N-7. The other two coordination sites are occupied by water molecules which are hydrogen bonded to O-6. This atom is not involved in a direct bonding to the metal. Two bonds to chloride ions are longer thus forming distorted centrosymmetrical octahedral molecules. In the second complex, (B), the coordination geometry at copper is square-pyramidal with two ACV molecules bound to N-7 in *trans*-basal positions. The other three coordination sites are occupied by aqua ligands. The oxygen atom of the carbonyl group (O-6) is neither involved in intramolecular hydrogen binding to the coordinated aqua ligands nor in binding to the metal. In the third complex, (C), copper is also bound to two ACV molecules but the geometry at the metal site is slightly distorted square planar. The other two coordination sites are occupied by aqua ligands. The ligand carbonyl oxygen O-6 is additionally weakly bound to the Cu(II) atom, and only in this complex a pseudo-chelate N-7/O-6 bonding of ACV to the copper was observed. It seems that the differences mentioned above, affect the relative cytotoxic level of tested Cu-ACV complexes [(C)>(B)>(A)]. However, during the cytotoxicity studies we showed that 20 - 40(g/ml ACV are able to stimulate the viability of MDBK cells. Moreover, this effect is well manifested between 48 and 72h in samples grown in the presence of the lowest concentration - 20(g/ml. Up to now, we did not find any evidence showing that low levels of ACV are able to stimulate cell viability. Additional experiments have to be performed to confirm these data in different cell types, as well as to explain the mechanism/s through which ACV exert this effect.

Secondly, the anti-HSV effect of Cu-ACV complexes was studied in seeded MDBK cells and compared to ACV used in the same concentration range. Calculating $IC_{50}$ values we checked also the relationship between the structure and anti-HSV activity of complexes. A viral determined effect was also under attention. For this purpose we used two strains sensitive to ACV - one from the first type of HSV (DA) and the second one Bja from HSV type 2. The third strain - R-100 (HSV-1) is resistant to ACV and its gene for TK is altered. This strain served as a sensitive control through which the modulation of anti-HSV activity of ACV being in a complex with Cu(II) was elucidated. The least effective against HSV replication in MDBK cells was complex (B) with square-pyramidal geometry at Cu(II) in which O-6 is neither involved in binding to three water ligands nor to the metal. Concerning (A) and (C) complexes, their inhibitory activity depend on the specificity of the particular viral strain. Thus, for ACV sensitive viruses DA (HSV-1) and Bja (HSV-2) the relative rates are: ACV ( (A) > (C) and (A) >> ACV ( (C) respectively. These data show that the complex (A), containing two Cl ions which binds to Cu(II) at rather longer distances, is a more potent inhibitor of HSV-2 than ACV. Moreover, the ACV resistant strain R-100, which TK substrate specificity is altered, is more sensitive to this complex than to ACV itself and the relative rate is: (A) >> ACV ( (C). Taking in mind the fact that to initiate the first step of transformation of ACV to the active form

- ACV-MP a viral specific enzyme TK is necessary to participate we propose that in the inhibition of the less sensitive to ACV viruses like Bja or ACV resistant ones, other (feed back) machanism/s is/are involved. The possibilities which we would like to propose are as follows: 1. A simultaneously occuring process of Cu-mediated DNA damage in virus-infected cells, as recently reported by Sagripanti et al. (1997); 2. Potentiation of the antiviral activity of ACV by chloride ions, which is clearly proven when chlorides are applied in combination with other compounds.

These findings are consistent with previously published data and undoubtedly show once again that Cu(II) complexes and Cu-ACV in particular, could be useful in the treatment of HSV infections, especially when the causative agent is a resistant to ACV mutant.

**Acknowledgements**

This work was partially supported by Bulgarian National Scientific Foundation, Project L-451.

**References**

1. Aruoma, O.I., Halliwell, B., Gajewski, E. and Dizdaroglu, M. *Biochem. J.*, 1991; **273**: 601-604.
2. Blazic, B., Turel, I., Bukovec, N., Bukovec, P. and Lazarini, F. J. *Inorg. Biochem.*, 1993; **51**: 737-744.
3. Chatis, P.A. and Crumpacker, C.S. *Antimicrob. Agents Chemother.*, 1992; **36**: 1589-1595.
4. Collins, P. *J. Antimicrob. Chemother.*, 1983; **12** (Suppl. B): 19-27.
5. De Clerq, E. *Antimicrob. Agents Chemother.*, 1982; **21**: 661-663.
6. Dizdaroglu, M., Rao, G., Halliwell, B. and Gajewski, E. *Arch. Biochem. Biophys.*, 1991; **285**: 317-324.
7. Elion, G.B., Furman, P.A., Fyfe, J.A., De Miranda, P., Beauchamp, L. and Schaeffer, H.J. *Proc. Natl. Acad. Sci. USA*, 1977; **74**: 5716-5720.
8. Elion, G.B. *J. Med. Virol.*, 1993; (Suppl. 1): 2-6.
9. Furman, P.A., St. Clair, M.H. and Spector, T. *J. Biol. Chem.*, 1984; **259**: 9575-9579.
10. Fyfe, J.A., Keller, P.M., Furman, P.A., Miller, R.L. and Elion, G.B. *J. Biol. Chem.*, 1978; **253**: 8721-8727.
11. Reardon, J.E. and Spector, T. *J. Biol. Chem.*, 1989; **264**: 7405-7411.
12. Sagripanti, J.L., Rouston, L.B., Bonifacino, A.C. and Lytle, C.D. *Antimicrob. Agents Chemother.*, 1997; **41**: 812-817.
13. Tilson, H.H., Engle, C.R. and Andrews, E.B. *J. Med. Virol.*, 1993; (Suppl. 1): 67-73.
14. Toyokuni, S. and Sagripanti, J.L. *Toxicol. Appl. Pharmacol.*, 1994; **126**: 91-97.
15. Turel, I., Bukovec, N., Goodgame, M. and Williams, D.G. *Polyhedron*, 1997; **16**: 1701-1706.
16. Varadinova, T., Shishkov, S., Bontchev, P., Nachev, C., Paskalev, Z., Strahilov, S., Toutekova, A. and Panteva, M. *J. Chemother.*, 1993; **5**: 3-9.
17. Varadinova, T., Shishkov, S., Panteva, M. and Bontchev, P. *Metal-Based Drugs* 1996; **3**: 149-154.

**Received: December 18, 1997 - Accepted: January 6, 1998 - Received in revised camera-ready format: January 12, 1998**

